# Let-7a mimic transfection reduces chemotherapy-induced damage in a mouse ovarian transplantation model

**DOI:** 10.1038/s41598-022-14926-z

**Published:** 2022-06-27

**Authors:** Chrysanthi Alexandri, Geraldine Van Den Steen, Isabelle Demeestere

**Affiliations:** Research Laboratory in Human Reproduction, Faculty of Medicine, Université Libre de Bruxelles (ULB), Brussels, Belgium

**Keywords:** Molecular medicine, Oncology

## Abstract

Pharmacological approaches offer a non-invasive and promising option for fertility preservation in young female cancer patients undergoing gonadotoxic therapy. The GnRH-agonists are the only clinically available drugs in this indication, but their use and mechanisms of protection are still controversial. Recently, we have investigated new targeted drugs based on microRNA (miRNA) replacement therapy, and have identified the let-7a miRNA as candidate for fertility preservation strategies. Here, the effect of let-7a replacement during chemotherapy exposure on follicular growth and oocyte maturation capacity was investigated using a mouse ovarian-kidney transplantation model. Newborn mouse ovaries were cultured under different conditions; control, chemotherapy exposure (4-hydroperoxycyclophosphamide, 4-HC), and co-treatment with 4-HC and let-7a mimic transfection (4-HC + let-7a). The ovaries were then transplanted under the kidney capsule of recipient mice and follicular growth, survival, and oocyte in vitro maturation were assessed after 3 weeks. The results showed that the follicular pool was highest in the control group but higher in the 4-HC + let-7a group than the 4-HC group. DNA-damage/apoptosis ratios were higher in all 4-HC-exposed groups compared to control but were reduced in the 4-HC + let-7a group. In addition, the post-transplantation oocyte in vitro maturation rate was higher in the 4-HC + let-7a group compared to the 4-HC group, suggesting better oocyte quality. These results provide new information regarding the beneficial effects of let-7a replacement against chemotherapy-induced ovarian damage and open new perspectives for future in vivo applications.

## Introduction

Standard oncological treatments such as chemotherapy or radiotherapy are effective for curing the majority of young patients diagnosed with cancer but can have a negative impact on long-term quality of life^1–3^. In young female cancer survivors, in particular, fertility and pregnancy-related morbidities induced by gonadotoxic oncological treatments are a high priority. Several scientific guidelines have highlighted the importance of counselling all young patients about their risk of premature ovarian failure (POF) and offering fertility preservation options. The mechanisms of chemotherapy-induced ovarian damage have been extensively studied and likely involve several pathways that affect multiple physiological processes such as growth, survival, and acquisition of reproductive competence, depending on the type of treatment^4^. The degree of gonadotoxicity associated with each type of treatment is determined by different parameters including the type and dose of the chemotherapeutic drug administered, and the patient’s age and ovarian reserve prior to treatment initiation^5^. Specifically, chemotherapeutic regimens that include alkylating agents such as cyclophosphamide (CPA) that have active metabolites considered to be highly gonadotoxic are often administered to treat young patients^6^. Therefore, there is an increasing interest in identifying new strategies to prevent this therapy-induced damage.

The established method for fertility preservation (FP) in adults and post-pubertal girls is oocyte and embryo cryopreservation after controlled ovarian stimulation^3^. However, this procedure has some limitations, including its invasiveness, the delay required for ovarian stimulation, and age-related issues as the method is not applicable in pre-pubertal patients. Immature oocytes can also be collected from antral follicles during natural cycles and in vitro matured (IVM) but the efficiency of this experimental technique in oncological patients appears to be limited^7^. Another innovative alternative is ovarian tissue cryopreservation which is currently the only available option for prepubertal and pubertal girls or when gonadotoxic treatment has already started^8^. However, the main restriction of this strategy is the risk of residual malignant cells, potentially present in cryopreserved ovarian fragments, that could induce cancer relapse after ovarian tissue transplantation^9^. In addition, ovarian pharmacological protection appears to be very attractive. Given the limitations of the new therapeutic options recently developed in this field^10,11^, novel and more advanced targeted FP strategies aiming to protect the ovaries during oncological treatment should be developed^12^.

In a previous study, we investigated the ovarian protective potential of microRNAs (miRNAs). MicroRNAs are small non-coding RNA molecules (~ 22 nucleotides) that act as major post-transcriptional gene regulators. The key role of miRNAs in ovarian function is well established^12,13^ and their unique characteristics make them attractive tools for FP strategies. Specifically, miRNAs are able to regulate chemo-sensitivity and chemo-resistance^14,15^. As their profile is modified during chemotherapy exposure, they could potentially act to minimize the side effects of anti-cancer therapies by reducing off-target toxicity. Moreover, studies based on next-generation RNA sequencing have revealed that different organs are characterized by a unique miRNA-signature, facilitating tissue-specific targeting^12^. Most importantly, as a single miRNA targets several genes, it potentially acts on the expression of a gene network that is activated during chemotherapy exposure, including key processes in the ovary such as apoptosis, DNA damage response, and ovarian follicle activation^16–18^.

Based on this evidence, we have demonstrated that the expression levels of several miRNAs are dysregulated during exposure to an active metabolite of cyclophosphamide, 4-hydroperoxycyclophosphamide (4-HC), in newborn mouse ovaries and, among them, let-7a was the most downregulated^19^. Let-7a belongs to the let-7 miRNA family that is highly conserved among species, and has tumor-suppressor activity depending on the type of tissue and genes targeted^20,21^. However, beyond the anti-apoptotic effect of let-7a in mouse postnatal ovaries during chemotherapy exposure and in porcine granulosa cells during follicle atresia, our knowledge about its function in mammalian ovaries is still limited^19,22^.

In this study, we further investigated the ovarian protective potential of let-7a during in vitro exposure to 4-HC in mouse newborn ovaries and evaluated the effects of a let-7a mimic replacement approach on subsequent follicular development and acquisition of oocyte maturation competence using an ovarian-kidney transplantation model.

## Results

### Let-7a replacement therapy protects mouse PND3 ovaries after 24 h exposure to 4-HC/20 µM in vitro

**Figure 1:**
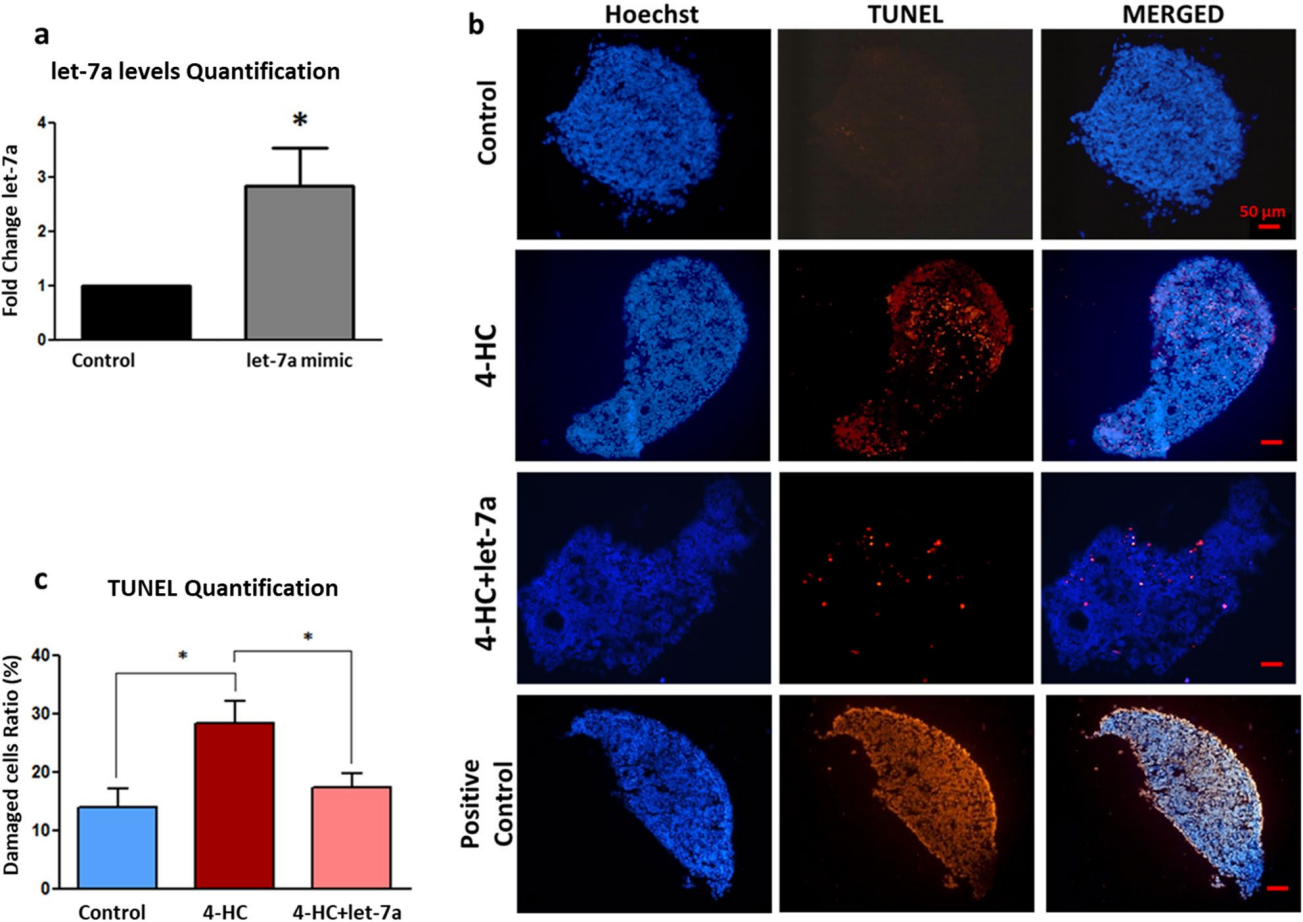
(**a**) Increased expression of let-7a in PND3 ovaries after transfection with let-7a mimic using liposome delivery system (N = 3). (**b**) DNA-damage/apoptosis assessment in PND3 ovaries after 2 days culture in three different conditions: control, 4-HC, and 4-HC + let-7a. Ovarian sections show nuclear labelling with Hoechst (blue) and DNA-damaged/apoptotic cells /TUNEL positive (red) (scale bar; 50 µm). The transfection with let-7a mimic reduced chemotherapy-induced damage. (**c**) The quantification of damaged cells was performed in at least three different ovarian sections (N ≥ 3) for each experimental group (N = 7). Each result is presented as the mean, the error bars represent the standard error, *p < 0.05.

**Figure 2:**
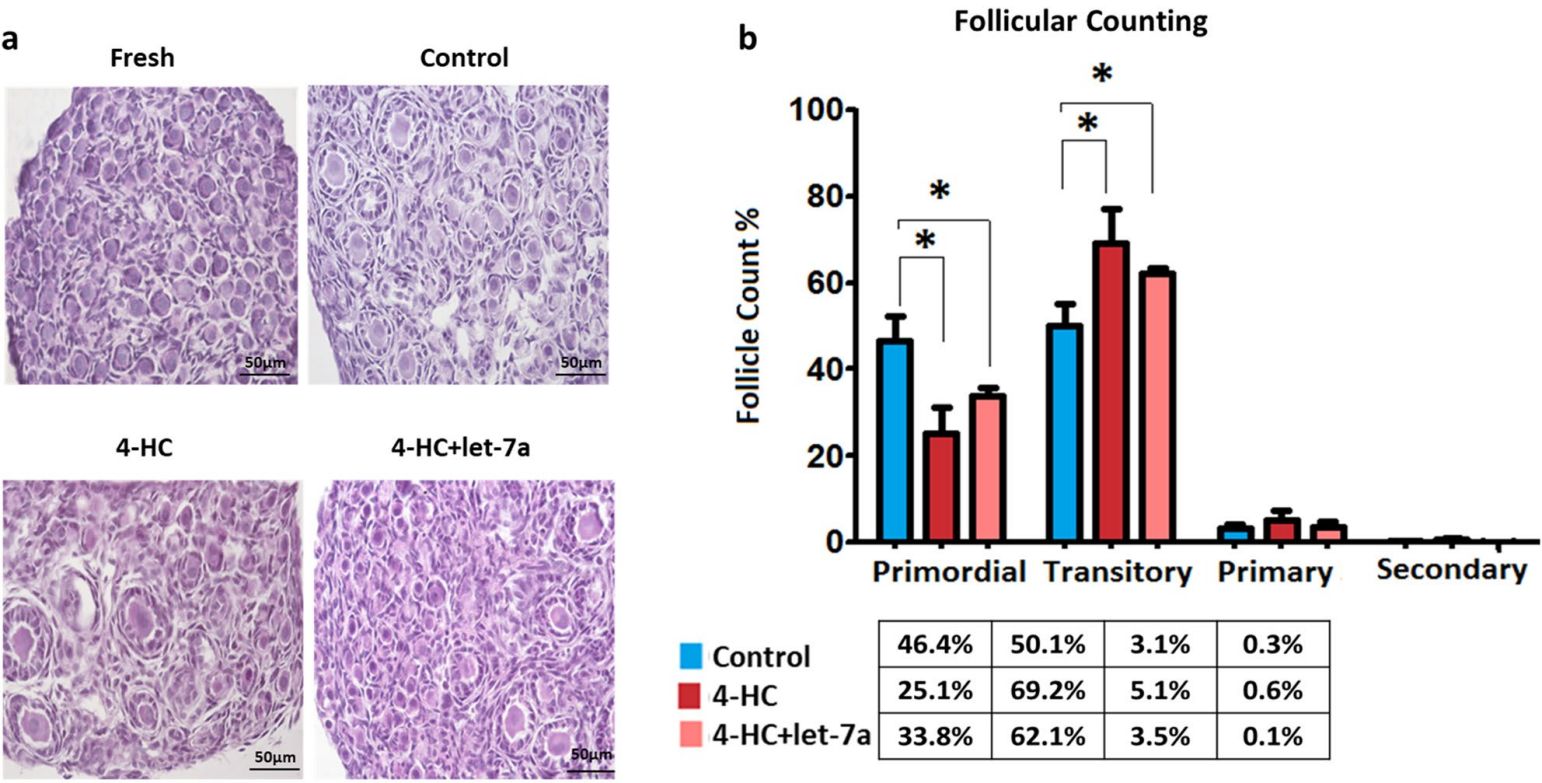
(**a**) Hematoxylin & Eosin (H&E) staining of PND3 ovaries in different conditions: D0 fresh ovary, cultured control, 4-HC, and 4-HC + let-7a mimic (N = 4/condition). (**b**) Percentage of follicles in different developmental stages in the 3 groups: control, 4-HC and 4-HC + et-7a mimic. The error bars represent the standard error, *p < 0.05.

First, we evaluated the efficacy of let-7a transfection in PND3 ovaries in vitro prior and concomitant to chemotherapy exposure using Lipofectamine RNAiMAX as previously described^19^. In order to confirm the effectiveness of the let-7a mimic transfection, the miRNA levels of let-7a were quantified by real-time PCR using individual TaqMan Assays. The results demonstrated a 2.9-fold increase in let-7a levels in the transfected ovaries compared to non-transfected ovaries (control group) (*p* = 0.037), confirming the success of the in vitro transfection (Fig. 1a). Next, DNA-damage/apoptosis evaluation by TUNEL assay demonstrated a significant reduction in chemotherapy-induced ovarian damage after combined let-7a transfection and 4-HC exposure compared to 4-HC exposure alone (17.41% vs 28.43% positive staining; *p* = 0.035) (Fig. 1b,c). After 24 h exposure to 4-HC, the number of primordial follicles was significantly reduced in the 4-HC group compared to the control group (25.1% vs 46.4%; *p* = 0.021). The proportion of primordial follicles that demonstrated follicular activation tended to be higher in the 4-HC + let-7a group compared to the 4-HC alone group (33.8% vs 25.1%, *p* = 0.24), although this result was not statistically significant and remained lower compared to controls (46.4%, *p* = 0.043). Accordingly, the proportion of transitory follicles was significantly higher in the 4-HC group but not in the 4-HC + let-7a group compared to controls, suggesting a slight effect of let-7a to prevent follicular activation induced by chemotherapy (Fig. 2a,b), (Supplementary Table, S1).

### Transfected PND3 mouse ovaries are able to grow 21 days post-transplantation

**Figure 3:**
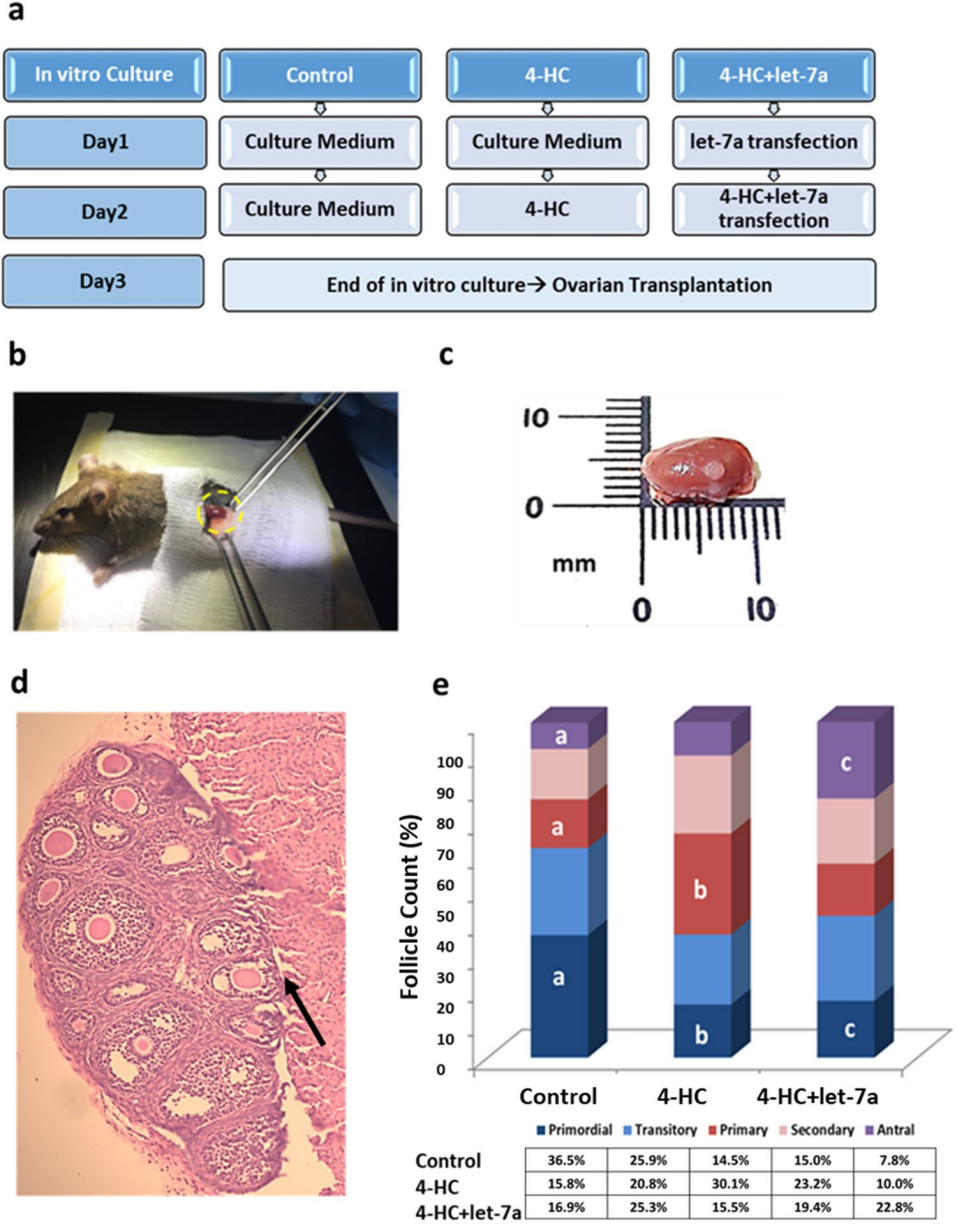
(**a**) Postnatal-day 3 (PND3) ovaries were cultured for 2 days under three different conditions: control, 4-HC, and 4-HC + let-7a. (**b**) Ovarian transplantation procedure in the kidney capsule of adult female mice. (**c**) Kidney collected at day 21 post-transplantation. (**d**) Arrow shows the PND3 ovaries. Hematoxylin & Eosin (H&E) staining on a section of grafted ovary, showing the fusion between the two organs. Follicles from different developmental stages were identified. (**e**) Percentage of follicles in different developmental stages (primordial; blue, transitory; light blue, primary; red, secondary; pink, antral; purple) in 3 groups (control, 4-HC, 4-HC + let-7a) in the transplanted ovaries (N = 3/condition). The error bars represent the standard error, the differences between the letters a-b, a-c for each type of follicle are statistically significant *p* < 0.05.

**Figure 4:**
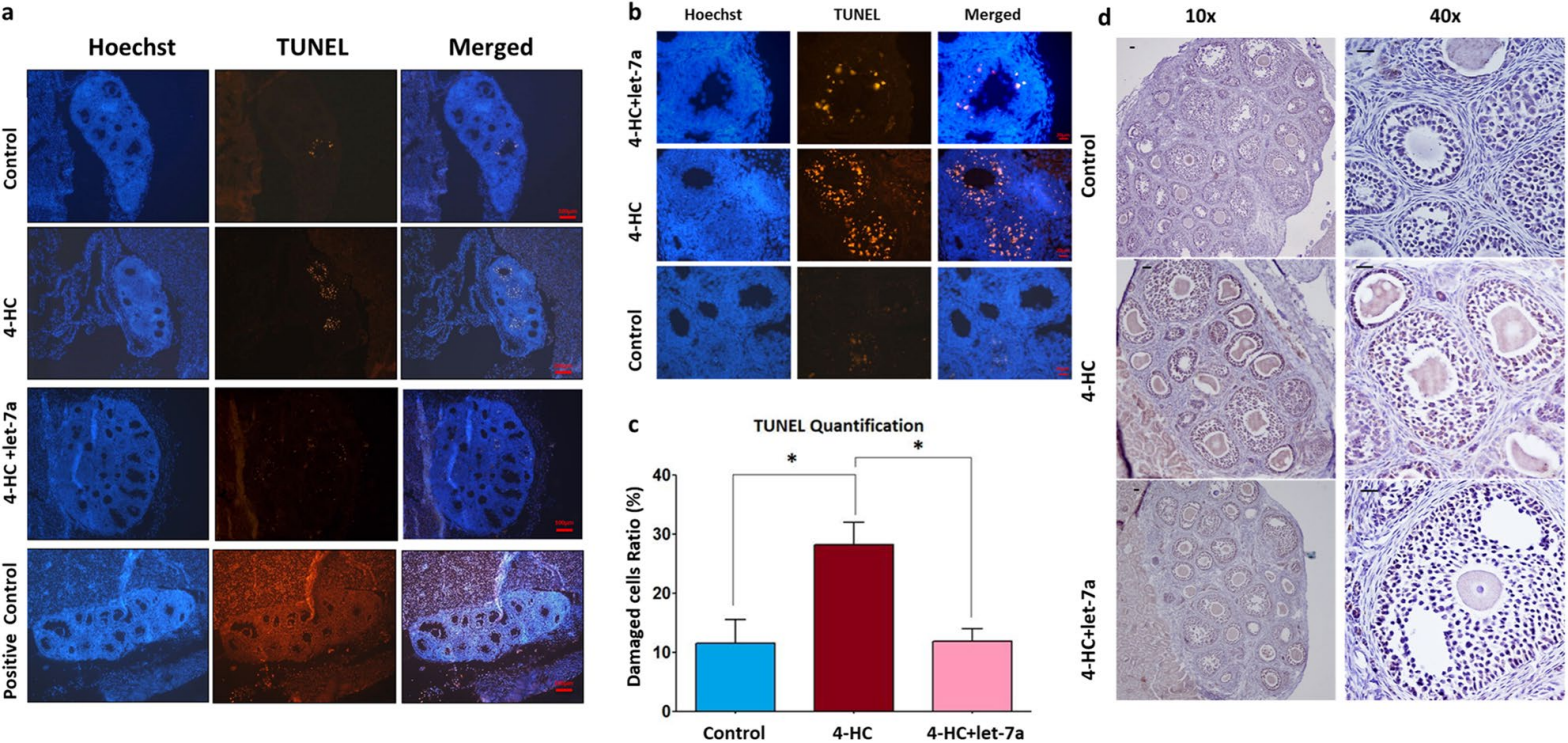
Evaluation of gonadotoxic damage in grafted ovaries in three different groups: control, 4-HC, and 4-HC + let-7a. (**a**) Immunofluorescent sections show nuclear labelling by Hoechst (blue) and DNA damaged/apoptotic cells by TUNEL (red), scale bar; 100 µm and (**b**) magnified images, scale bar; 20 μm. (**c**) The quantification of damaged cells was performed in at least three different ovarian sections (N ≥ 3) for each experimental group (N = 3). The error bars represent the standard error, *p < 0.05. (**d**) Hematoxylin & Eosin counterstaining and immunostaining of FAS receptor on 5 µm sections of grafted ovaries in different conditions (N = 2). The apoptotic cells of the ovarian follicles are FAS positively stained, (10×) and magnified images (40×), scale bar; 20 μm.

After 48 h of in vitro culture under three different conditions (Fig. 3a), PND3 ovaries were transplanted under the kidney capsule of adult female mice (Fig. 3b). At day 21 post-transplantation, the mice were sacrificed and follicular development, count, and apoptosis were evaluated. Macroscopically, the ovaries were well vascularized and grown under the capsule (Fig. 3c,d). H&E staining revealed that follicles from different developmental stages were present in all conditions (control, 4-HC, 4-HC + let-7a) (Fig. 3e). The proportion of primordial follicles was significantly lower in the 4-HC (15.8%) and 4-HC + let-7a (16.9%) groups compared to the control group (36.5%) (*p* = 0.014, *p* = 0.034), while the number of antral stage follicles was significantly higher in the 4-HC + let-7a group (22.8%) compared to the control group (7.8%) (*p* = 0.034) (Fig. 3e). In contrast to in vitro experiments, these results suggested that let-7a mimic transfection did not reduce subsequent follicle activation and growth after transplantation. However, the TUNEL assay demonstrated a lower apoptosis rate in the 4-HC + let-7a group compared to the 4-HC group (11.6% vs 28.2%, *p* = 0.034) (Fig. 4a–c). This observation is also confirmed by the immunohistochemistry experiments for FAS receptor (surface antigen APO1 or CD95)-a member of the tumor-necrosis receptor family which can induce apoptosis by stimulatory signals. Specifically, the staining intensity for the FAS receptor in the 4-HC group appears higher compared to 4-HC + let-7a group while the intensity is much lower in the control group (Fig. 4d).

### Let-7a transfection has a beneficial effect on oocyte maturation capacity in vitro

**Figure 5:**
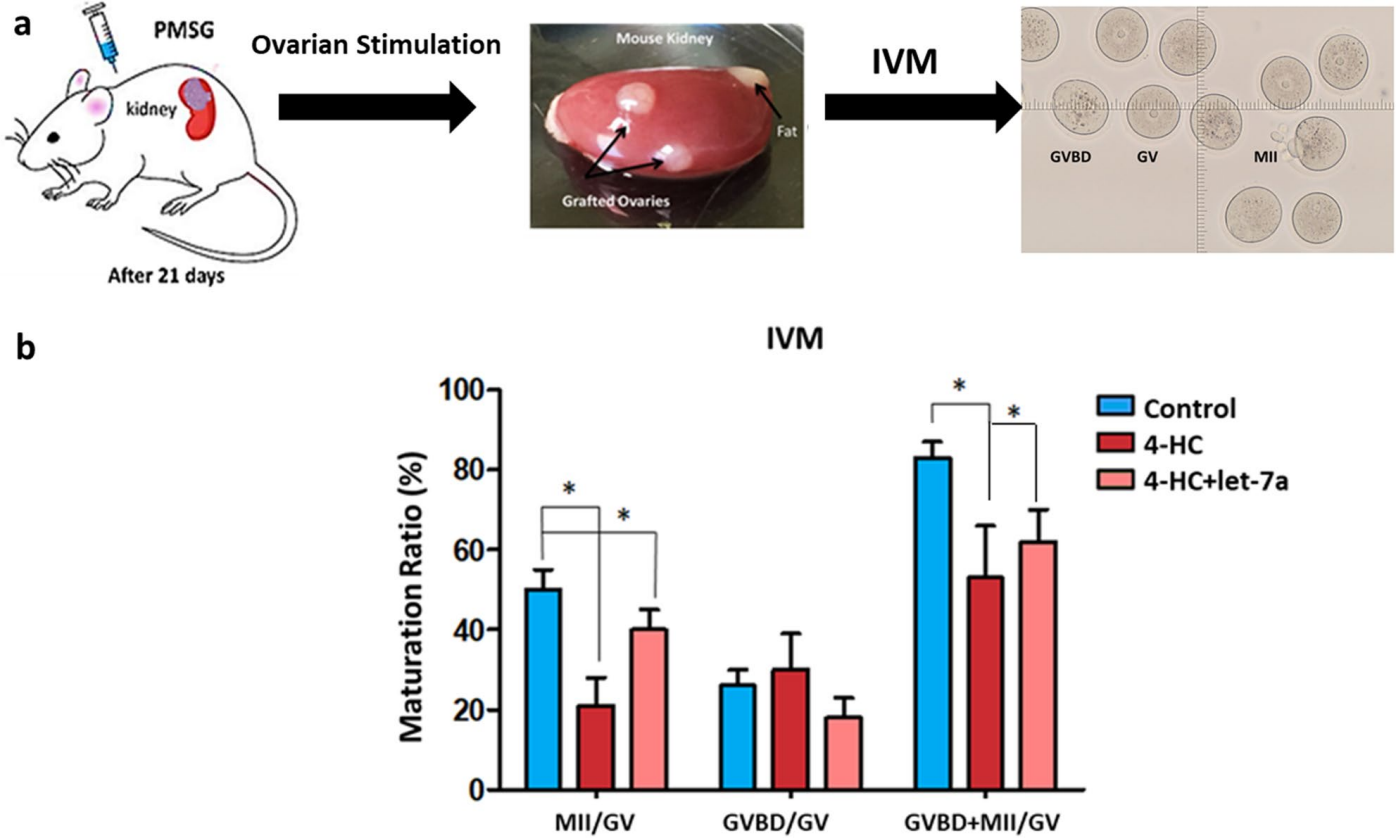
Oocyte maturation after three different types of treatment: control, 4-HC, and 4-HC + let-7a. (**a**) At day 21 post-transplantation, mice were injected with PMSG and grafted ovaries were collected to mechanically isolate OCC. The germinal vesicle (GV) oocytes were matured for 24 h before evaluation of the maturation rate (**b**) The number of GV oocytes that underwent maturation to germinal vesicle break down (GVBD) and metaphase II (MII) stage after 24 h in maturation medium were calculated and the maturation ratio was expressed as a percentage. The results are presented as the mean of at least 7 different experimental samples for each group (N ≥ 7). The error bars represent the standard error, *p < 0.05.

Three weeks post-transplantation, the recipient mice were injected intraperitoneally with 100 µl of 5 IU pregnant mare serum gonadotropin (Gentaur Molecular Products, Belgium) to induce ovarian stimulation. The following day, the ovarian grafts were removed from the kidney after euthanasia by cervical dislocation (Fig. 5a). The OCC were mechanically collected and matured in vitro for 24 h. Oocyte maturation stages were categorized as germinal vesicle (GV), germinal vesicle break down (GVBD) or metaphase II (MII) (Fig. 5a). The results demonstrated an improvement in MII/GV rate after 24 h in the 4-HC + let-7a group compared to the 4-HC alone group (40% vs 21%, *p* = 0.027), although it remained lower compared to controls (50%, *p* = 0.19) (Fig. 5b).

## Discussion

These results confirm our previous findings suggesting an ovarian protective effect of let-7a against the cytotoxic effects of chemotherapy in vitro, and demonstrate that this protective effect remains after in vivo follicular development.

The condition of ovarian toxicity was induced by treatment with 4-hydroperoxycyclophosphamide (4-HC) for 1-24 h. Given that cyclophosphamide is an inactive prodrug that needs in vivo activation, the 4-HC was used as a pre-activated analogue to cause chemotherapy-induced ovarian damage. The evaluation of cyclophosphamide metabolites’ toxicity on cultured mouse ovaries has revealed that the 4- HC was the most gonadotoxic because its high hydrophobicity facilitates the cellular entrance and generation of phosphoramide mustards which finally cause the damage (PM)^23^. According to the results from the study of Horicks et al., when isolated ovarian follicles were exposed to 4-HC (20 μM), both follicular survival and oocyte maturation rate were significantly reduced^24^. Moreover, this dose was tested on PN3 ovaries showing apoptosis without depletion of the whole ovarian reserve^25^. Hence, in the current study, this concentration was selected to be tested in vitro for inducing damage to PND3 ovaries. As the goal of the study is to monitor the early response of miRNAs to the changes of their microenvironment and their possible implication in pathways such as apoptosis, DNA damage response and follicle activation, the chemotherapy dosage should cause a toxic effect without destroying the follicles completely by inducing irreversible necrosis.

Apoptosis assessment revealed that the miRNA-mimic could significantly reduce the cell-damaging effects of the chemotherapy even after 24 h in vitro exposure in PND3 ovaries. In addition to the effect on apoptosis, follicular activation constitutes another mechanism of gonadotoxicity as it accelerates the recruitment of primordial follicles and ovarian reserve depletion^26^. The assessment of follicle activation confirmed that chemotherapy induced an increase in transitory follicles in vitro while transfection with let-7a tended to slow down this effect, although the difference was not significant. This moderate effect on follicular activation was previously suggested after short-term exposure^19^.

In vivo experiments using an ovarian transplantation model allowed the evaluation of the subsequent follicular growth and survival. The kidney was selected as a site for ovarian tissue transplantation because it is well vascularized and offers an efficient blood supply to support graft healing and viability^27^. The surrounding kidney capsule can be expanded in order to accommodate the transplanted ovary and to allow growth of the grafted organ^28^. The transplanted ovaries remained in the kidney capsule for 3 weeks, which is the estimated time required for an ovarian follicle to be developed from the primordial to antral stage in mouse models^29^. Using this model, we demonstrated the benefit of let-7a transfection during chemotherapy exposure on in vivo follicular development and oocyte competence. Follicle percentages revealed that in vitro exposure to chemotherapy for 24 h had a long-term impact on the ovarian reserve by reducing the number of primordial follicles compared to control conditions. Transfection with let-7a prior and concomitantly to chemotherapy exposure reduced apoptosis in growing follicles but did not prevent quiescent follicular activation compared to control. It is well-know that massive activation also occurs in vivo after transplantation^30^ and this effect is probably exacerbated due to prior chemotherapy exposure^25^. By protecting the growing follicles exposed to co-treatment (4-HC + let-7a), a higher number of growing follicles may reach the antral stage compared to control, although the ability of the oocytes to mature in vitro remains lower. In the 4-HC group, there were less antral follicles as the toxic effects of the treatment induced follicular death in earlier developmental stages and impact further oocyte maturation process. Further experiments should be performed to evaluate the long-term benefit of this strategy by assessing the ability of these oocytes to be fertilized after longer in vivo development. However, a possible explanation for this observation could be the effect of bidirectional communication between the oocyte and granulosa cells that supports survival, growth, and differentiation of both cell-types. Specifically, tight interactions between the oocyte and granulosa cells in primordial follicles is further facilitated by trans-zonal projections and gap junctions when the physical barrier of the zona pellucida appears at the secondary stage^31^. Throughout folliculogenesis, a continuous exchange of nutrients (cholesterol, pyruvate, amino acids), growth factors (growth differentiation factor 9 [GDF9], bone morphogenetic protein 15 [BMP15], fibroblast growth factor 8b [FGF8B]) and other small molecules occurs between oocytes and granulosa cells^32^. More recently, the presence of membrane structured extracellular vesicles (EVs) has been identified in ovarian follicular fluid. The EVs contain not only mRNAs and proteins, but also miRNAs, indicating an alternative pathway for intra-follicular communication^33,34^. According to a study in mare follicular fluid, microvesicles and exosomes are enriched in miRNAs and microvesicle-uptake was observed in granulosa cells. Moreover, gene expression analysis revealed a decrease in the expression of potential miRNA targets in granulosa cells after transfection of these vesicles. Bioinformatics analysis showed that these targets participate in mitogen-activated protein kinase (MAPK) signaling, focal adhesion, and the transforming growth factor beta (TGFB) pathway^33^. Therefore, we can hypothesize that the initial transfection with let-7a mimic helps to restore the levels of let-7a in granulosa cells and, hence, protects primordial/primary follicles from apoptosis after exposure to a gonadotoxic metabolite of cyclophosphamide. Therefore, the improved survival of granulosa cells, which are the nursing cells for the oocyte, could indirectly lead to oocyte protection. Nevertheless, the regulation mechanism of let-7a should be further addressed as well as the identification of its targets and function in the ovary. Scientific evidence has demonstrated that several feedback loops exist between let-7 and regulatory factors such as Fas-Dicer-let-7-Fas with an important role in apoptosis^35^. The let-7a recognizes the 3-UTR of the targeted mRNA but it can also bind to other sites, including coding regions that inhibit mRNA translation into proteins. In our previous study, we demonstrated that let-7a restoration was able to reduce the levels of Fas ligand (FasL) mRNA during chemotherapy exposure in mouse ovaries but large-scale target analysis is required to elucidate the anti-apoptotic mechanism of let-7a^19^. Moreover, regarding the oncogenic activity of the let-7a, it has been demonstrated that let-7-family members are implicated in cancer but as tumor suppressor genes. However, the role of let-7 family in proliferation, invasion and apoptosis of cancer cells is highly depended on the tissue and time of expression. Characteristically, it has been shown that let-7 can prevent the proliferation of cancer cells by targeting oncogenes like rat sarcoma (RAS), high‐mobility group AT‐look 2 (HMGA2), c‐Myc, Janus protein tyrosine kinase (JAK), signal transducer and activator of transcription 3 (STAT3)^36^.

Despite the encouraging results derived from this in vivo approach, there are important limitations that should be taken into consideration. The first limitation concerns the bias introduced by the several in vitro and in vivo steps before the final evaluation of oocyte quality. The in vitro culture of the PND3 ovaries as well as the transplantation procedure can induce spontaneous follicle activation regardless of the treatment. Moreover, the transplantation process can also introduce bias due to manipulation procedures^37,38^. To overcome these limitations, a new methodology for in vivo administration of the let-7a mimic will be developed using an appropriate delivery system to achieve efficient and targeted transfection^12^. The liposomes used for in vitro transfection are not appropriate for targeted miRNA transfer into the ovaries and pose low stability and limited organ-specificity in vivo. The data on in vitro oocyte fertilization rates, blastocyst formation, and embryo development are needed to confirm the benefit on oocyte quality. The ultimate goal of this study is to obtain healthy live offspring and to assess the safety of the treatment (miRNA mimic transfection, chemotherapy) initially applied to the ovaries and to develop a novel miRNA-delivery system for in vivo approaches.

However, the development of an efficient miRNA-delivery system with transfection capacity, minimal cytotoxicity and specific-organ targeting is quite challenging. While viral and non-viral (i.e. liposomes) vectors have been widely studied as gene-transporters, they still pose a lot of limitations including instability in blood circulation, triggering of inflammation and immune responses. Given these drawbacks, our interest is focused on gold nanoparticles (AuNPs) as novel and promising miRNA-vectors^12^. There is an increasing interest in the use of AuNPs because they are biocompatible and they can be easily synthesized or functionalized with various biomolecules. Hence, the delivery of the let-7a mimic into the ovaries can be achieved by active ‘’drug’’ targeting; an approach that involves interactions between ‘’biological pairs’’ (receptor-ligand, antibody-antigen, enzyme-substrate). Therefore, functionalized AuNPs would offer the opportunity to attach different ligands at their surface in controlled densities, i.e. one miRNA and one ovarian-specific ligand allowing selective delivery into ovarian cells.

Regarding the future in vivo studies in animal models, the ovaries of anesthetized adult mice can be injected with the functionalized AuNPs. AuNPs-let-7a mimic conjugates will be injected prior and concomitantly to chemotherapy. At the end of the treatments, the mice will be sacrificed and the possible protective effect of AuNPs conjugates on the ovaries will be evaluated. In terms of clinical application in humans, it can be hypothesized that the proper functionalization of the AuNPs will allow the intravenous administration of the miRNA-based drug and offer the opportunity for ovarian specific targeting avoiding the interaction with other tissues, the off-target toxicity or the interference with cancer treatment. Certainly, the transition from bench to bedside is a complex procedure and requires multiple steps of safety assessment at the preclinical level (Supplementary Table S1).

## Methods

### Animal model

C57BL/6xCBAF1 female hybrid mice were used for all the experiments. Newborn mice, at day 3 after birth (PND3), were used for in vitro cultures and adult female recipient mice (~ 1–3 months old) were used for the in vivo ovarian transplantation experiments. The animals were maintained under standard monitored light and temperature conditions and they had access to food and water ad libitum.

### In vitro culture of PND3 mouse ovaries

Female newborn mice (PND3) were sacrificed by decapitation and ovaries were carefully harvested and collected in Leibovitz L-15 medium (Life Technologies, Belgium) supplemented with fetal bovine serum (FBS 10%), 1 mg/ml streptomycin, and 6 mg/ml penicillin G (Sigma, Belgium). Whole ovaries were separated from the surrounding tissues and placed in culture plates. Each ovary was cultured on polycarbonate inserts using Corning® 24-Well Tissue Culture-Treated Plates, in 400 μl Opti-MEM reduced-serum medium (Invitrogen, Life Technologies Europe BV) supplemented with ascorbic acid (50 μg/ml), human transferrin (27.5 μg/ml), penicillin G (5 IU/ml), and streptomycin sulphate (3.7 IU/ml) (Sigma, Belgium). The ovaries were cultured at 37 °C in a humidified incubator with 5% CO_2_^19^. In order to evaluate the effects of chemotherapy exposure, the PND3 ovaries were cultured in the presence or absence of 4-hydroperoxycyclophosphamide (4-HC, 20 μM) (Sigma, Belgium) at day 2 for 24 h. To evaluate the effects of miRNA mimic transfection, the PND3 ovaries were cultured in the presence of let-7a mimic (300 pmol) from day 1 for 48 h^19^. The transfection was performed prior and concomitant to chemotherapy exposure, following the methodology previously described^19^. All ovarian samples were processed for analysis at Day 3 of culture. Three conditions were tested: culture alone (control), chemotherapy exposure for 24 h (4-HC), and chemotherapy exposure for 24 h + let-7a mimic (4-HC + let-7a mimic).

### Individual TaqMan® gene expression assays

The let-7a expression levels in PND3 ovaries, control, and mimic-transfected (N = 3) were evaluated by real-time PCR with individual TaqMan® primer-probes. For each experiment, PND3 ovaries were pooled from 2 mice from the same litter and randomly assigned into control and let-7a mimic transfected groups. The RNA extraction focused on small non-coding RNA molecules from PND3 ovaries was performed using ReliaPrep™ miRNA Cell and Tissue Miniprep System (Promega, Netherlands) according to the manufacturer’s instructions including homogenization and DNAse treatment steps. The quantity and purity of RNA was determined using a NanoDrop spectrophotometer (Thermo Scientific, Belgium). Then, miRNA samples were processed for cDNA synthesis using the TaqMan® MicroRNA Reverse Transcription kit (Applied Biosystems, Belgium) and multiplexed Megaplex™ RT Primers (Applied Biosystems™) according to the manufacturer’s instructions. A step of pre-amplification was performed using 2.5 µl from the cDNA product, TaqMan® PreAmp kit (Applied Biosystems, Belgium) and Megaplex™ PreAmp Primers (Applied Biosystems, Belgium) according to the manufacturer’s instructions. The pre-amplified products were diluted in a final volume of 100 µl Tris–EDTA buffer (pH 8). Briefly, 1 µl from the diluted pre-amplified product was combined with 1 µl of TaqMan™ MicroRNA Assay (20x) (Applied Biosystems, Belgium) and 10 µl of TaqMan™ PCR Master Mix (Applied Biosystems, Belgium) in a final volume of 20 µl. The expression level of let-7a was assessed using real-time PCR with individual TaqMan® primer-probes for the following: let-7a and endogenous controls U6, snoRNA202. All reactions were run in triplicate.

### Transplantation of newborn mouse ovaries

Adult, female recipient mice were anesthetized with intraperitoneal injection of 100–120 μl/20 g [100 μl Rompun 2% and 200 μl Ketamine (100 mg/ml) diluted in 700 μl physiological saline solution]. The surgery was performed under a stereoscope. After small dorso-horizontal incision under aseptic conditions, one of the two kidneys was gently exteriorized and a pocket-shaped opening was created in the kidney capsule using fine-end forceps. One or two in vitro cultured PND3 ovaries were placed underneath the capsule of the kidney.

### Histological studies and follicle counting

Three weeks after transplantation, the transplanted ovaries were collected with a part of the kidney and fixed in 4% paraformaldehyde for 24 h at 4 °C, embedded in paraffin and serially sectioned. Every fifth section from serially-sectioned ovaries was stained with hematoxylin and eosin (H&E) for assessment of follicular stage and count. The follicles were classified according to Gougeon’s criteria as primordial, transitional, primary, secondary, and antral^39^. Only follicles with a visible nucleus were counted.

### Immunohistochemistry

The FAS immunostaining was used to evaluate the apoptosis in the ovarian follicles. Fixed ovarian sections were deparaffinized and rehydrated in order to evaluate the follicular apoptosis (FAS) in mice ovaries. Antigen retrieval by heat was performed in citrate buffer pH6.0. The endogenous peroxidases were inhibited by hydrogen peroxide 1% (Merck Millipore, Belgium). Non-specific sites were blocked by normal goat serum and by Avidin/Biotin (Streptavidin/Biotin Blocking Kit Vector laboratories, United States) and the slides were incubated with the primary antibody (FAS (CD95) Invitrogen #PA5-79236) overnight at 4 °C. Then the sections were incubated in secondary antibody (goat anti rabbit biotinylated, Vector Laboratories #BA1000) and processed using an ABC kit according to manufacturer’s instructions (Vectastain Elite ABC systems, Vector Laboratories, United States). The reaction was revealed with diaminobenzidine (DAB Peroxidase Substrate Kit, Vector Laboratories, United States) followed by counterstaining with haematoxylin. As a negative control, primary antibodies were replaced by species-adapted non-specific IgG. Sections were examined using a Nikon Eclipse 50i fluorescent microscope.

### TUNEL assay

Apoptosis evaluation was performed by TUNEL staining (In Situ Death Cell Detection Kit, Roche, Belgium). Briefly, the ovarian sections were deparaffinized and rehydrated, and a proteinase K treatment was applied to make them permeable for the next steps (20 μg/ml in Tris 10 mM pH 7.4, Qiagen, Netherlands). A positive control was created by DNAse treatment. After a washing step, sections were labelled with TUNEL reagents according to manufacturer’s instructions (Roche) and counterstained with Hoechst (1 μg/ml) (Thermo Scientific, Belgium). Sections were observed using a Leica DM 2000 fluorescent microscope. The images were analyzed using ZEN 2.3 software on at least 3 randomly selected sections per ovary from 3 individual experiments. A lower threshold was used to minimize the background signal contribution and to exclude the auto-fluorescence signals. The Hoechst and TUNEL positive cells were quantified. The apoptosis level was estimated as the percentage of TUNEL-positive cells from the total Hoechst-positive cells per ovary.

### Oocyte in vitro maturation (IVM)

Three weeks following the transplantation, the recipient mice were injected intraperitoneal with 100 μl of pregnant mare serum gonadotropin (PMSG) (5 IU/100 μl) to induce ovarian stimulation and the day after, the mice were sacrificed by cervical dislocation. Then, the grafted ovaries were removed from the kidney and separated from the surrounding tissues with Leibovitz L-15 medium (Life Technologies, Belgium) supplemented with fetal bovine serum (FBS 10%), 1 mg/ml streptomycin and 6 mg/ml penicillin G (Sigma, Belgium). The oocyte-cumulus complexes (OCCs) were mechanically isolated from the grafted ovaries using needles. The OCCs were incubated in a maturation medium consisting of culture medium 5% ITS (MEM Alpha Medium-GlutaMAX (Life Technologies, Belgium)) supplemented with 5% fetal bovine serum (Life Technologies, Belgium), ITS (0.2 μl/ml, Sigma-Aldrich, Belgium), FSH (100 mIU/ml, Gonal-114 F®, Merck, Belgium), luteinizing hormone (LH, 10 mIU/ml, Sigma-Aldrich, Belgium), human chorionic gonadotropin (hCG, Pregnyl-Organon, 1.5 UI/ml), and epidermal growth factor (EGF 5 ng/ml) at 37 °C in a humidified incubator with 5% CO_2_. The oocytes were categorized as germinal vesicle (GV), germinal vesicle break down (GVBD) and metaphase II (MII). After 24 h, the maturation rate expressed as percentage of GVBD or MII obtained over the total of GV collected was calculated.

### Statistical analysis

All statistical analyses were performed by the IBM® SPSS Statistics 24 program. At least three biological replicates were performed for every experiment and all values represented as the mean or geometric-mean ± standard error of the mean (SEM). Non-parametric tests including the Mann Whitney U and Kruskal Wallis test were used to analyze non-normally distributed data among groups. *p* value < 0.05 was defined as a statistically significant difference.

### Ethics statement

All the experiments were approved by the local Animal Ethics Committee of “Université Libre de Bruxelles” and were performed according to ARRIVE guidelines and relevant regulations.

## Supplementary Information

Supplementary Table S1.

## Data Availability

For more clarity in the understanding and interpretation of the different experimentations, all data are available on request by contacting the corresponding author.

## References

[1] Siegel RL, Miller KD, Jemal A (2018). Cancer statistics, 2018. CA Cancer J. Clin..

[2] Lambertini M (2020). Fertility preservation and post-treatment pregnancies in post-pubertal cancer patients: ESMO Clinical Practice Guidelines†. Ann. Oncol..

[3] Anderson RA (2020). ESHRE guideline: Female fertility preservation†. Hum. Reprod. Open.

[4] Spears N (2019). Ovarian damage from chemotherapy and current approaches to its protection. Hum. Reprod. Update.

[5] Morgan S, Anderson RA, Gourley C, Wallace WH, Spears N (2012). How do chemotherapeutic agents damage the ovary?. Hum. Reprod. Update.

[6] Emadi A, Jones RJ, Brodsky RA (2009). Cyclophosphamide and cancer: Golden anniversary. Nat. Rev. Clin. Oncol..

[7] Fisch B, Abir R (2018). Female fertility preservation: Past, present and future. Reproduction.

[8] Demeestere I (2006). Ovarian function and spontaneous pregnancy after combined heterotopic and orthotopic cryopreserved ovarian tissue transplantation in a patient previously treated with bone marrow transplantation: Case report. Hum. Reprod..

[9] Dolmans M-M, Luyckx V, Donnez J, Andersen CY, Greve T (2013). Risk of transferring malignant cells with transplanted frozen-thawed ovarian tissue. Fertil. Steril..

[10] Li F (2014). Sphingosine-1-phosphate prevents chemotherapy-induced human primordial follicle death. Hum. Reprod..

[11] Kalich-Philosoph L (2013). Cyclophosphamide triggers follicle activation and “Burnout” AS101 prevents follicle loss and preserves fertility. Sci. Transl. Med..

[12] Alexandri C, Daniel A, Bruylants G, Demeestere I (2020). The role of microRNAs in ovarian function and the transition toward novel therapeutic strategies in fertility preservation: From bench to future clinical application. Hum. Reprod. Update.

[13] O’Brien J, Hayder H, Zayed Y, Peng C (2018). Overview of MicroRNA biogenesis, mechanisms of actions, and circulation. Front. Endocrinol..

[14] Zhao H (2016). MiR-770-5p inhibits cisplatin chemoresistance in human ovarian cancer by targeting ERCC2. Oncotarget.

[15] Blower PE (2008). MicroRNAs modulate the chemosensitivity of tumor cells. Mol. Cancer Ther..

[16] Zhang J, Xu Y, Liu H, Pan Z (2019). MicroRNAs in ovarian follicular atresia and granulosa cell apoptosis. Reprod. Biol. Endocrinol..

[17] Hatano K (2015). A functional screen identifies miRNAs that inhibit DNA repair and sensitize prostate cancer cells to ionizing radiation. Nucleic Acids Res..

[18] Tesfaye D (2018). MicroRNAs: tiny molecules with a significant role in mammalian follicular and oocyte development. Reproduction.

[19] Alexandri C (2019). MicroRNA profiling and identification of let-7a as a target to prevent chemotherapy-induced primordial follicles apoptosis in mouse ovaries. Sci. Rep..

[20] Pasquinelli AE (2000). Conservation of the sequence and temporal expression of let-7 heterochronic regulatory RNA. Nature.

[21] Boyerinas B, Park S-M, Hau A, Murmann AE, Peter ME (2010). The role of let-7 in cell differentiation and cancer. Endocr. Relat. Cancer.

[22] Cao R (2015). Expression and preliminary functional profiling of the let-7 family during porcine ovary follicle atresia. Mol. Cells.

[23] Desmeules P, Devine PJ (2006). Characterizing the ovotoxicity of cyclophosphamide metabolites on cultured mouse ovaries. Toxicol. Sci..

[24] Horicks F, Van Den Steen G, Gervy C, Clarke HJ, Demeestere I (2018). Both in vivo FSH depletion and follicular exposure to Gonadotrophin-releasing hormone analogues in vitro are not effective to prevent follicular depletion during chemotherapy in mice. MHR Basic Sci. Reprod. Med..

[25] Devos M, Grosbois J, Demeestere I (2020). Interaction between PI3K/AKT and Hippo pathways during in vitro follicular activation and response to fragmentation and chemotherapy exposure using a mouse immature ovary model. Biol. Reprod..

[26] Sonigo C, Beau I, Binart N, Grynberg M (2019). The impact of chemotherapy on the ovaries: Molecular aspects and the prevention of ovarian damage. Int. J. Mol. Sci..

[27] Li J (2010). Activation of dormant ovarian follicles to generate mature eggs. Proc. Natl. Acad..

[28] Robertson NJ, Fairchild PJ, Waldmann H (2007). Ectopic transplantation of tissues under the kidney capsule. Methods Mol Biol..

[29] Pedersen T (1970). Determination of follicle growth rate in the ovary of the immature mouse. J. Reprod. Fertil..

[30] Gavish Z (2018). Follicle activation is a significant and immediate cause of follicle loss after ovarian tissue transplantation. J. Assist. Reprod. Genet..

[31] Clarke HJ (2018). Regulation of germ cell development by intercellular signaling in the mammalian ovarian follicle. Wiley Interdiscip. Rev. Dev. Biol..

[32] El-Hayek S, Clarke HJ (2016). Control of oocyte growth and development by intercellular communication within the follicular niche. Results Probl. Cell Differ..

[33] Da Silveira JC, Veeramachaneni DNR, Winger QA, Carnevale EM, Bouma GJ (2012). Cell-secreted vesicles in equine ovarian follicular fluid contain miRNAs and proteins: A possible new form of cell communication within the ovarian follicle1. Biol. Reprod..

[34] Da Silveira J (2018). Isolation and analysis of exosomal microRNAs from ovarian follicular fluid. Methods Mol. Biol..

[35] Wang S (2011). Let-7/miR-98 regulate Fas and Fas-mediated apoptosis. Genes Immun..

[36] Wang X (2012). Regulation of let-7 and its target oncogenes (review). Oncol. Lett..

[37] Kawamura K (2013). Hippo signaling disruption and Akt stimulation of ovarian follicles for infertility treatment. Proc. Natl. Acad. Sci. USA.

[38] Grosbois J, Devos M, Demeestere I (2020). Implications of nonphysiological ovarian primordial follicle activation for fertility preservation. Endocr. Rev..

[39] Gougeon A (1996). Regulation of ovarian follicular development in primates: Facts and hypotheses. Endocr. Rev..

