# Supplementary material for: Let-7a mimic transfection reduces chemotherapy-induced damage in a mouse ovarian transplantation model
Source: Sci Rep. 2022 Jun 27;12:10863. doi: 10.1038/s41598-022-14926-z (PMC9237019; doi:10.1038/s41598-022-14926-z)
Supplement: Supplementary file 1 — Supplementary Table S1. [file 41598_2022_14926_MOESM1_ESM.pdf]

Supplementary Table S1: Counting of the follicle number in different conditions of ovarian culture

Follicle Counting Ovarian Culture	Primordial	Transitory	Primary	Secondary	Total
**4-HC 24h**	153	158	6	0	317
	81	82	9	1	173
	7	81	0	0	88
	299	150	16	0	465
**Mean**	135	118	8	0	261
**STDEV**	125	42	7	1	166
**4-HC+let7a**	196	101	0	0	297
	174	126	6	0	306
	194	264	18	3	479
	27	66	4	0	97
**Mean**	148	139	7	1	295
**STDEV**	81	87	8	2	156
**Control**	310	172	4	1	487
	208	221	8	0	437
	154	234	22	1	411
	72	164	9	0	245
**Mean**	186	198	11	1	395
**STDEV**	100	35	8	1	105
